# Application of the international classification of functioning, disability, and health to disability assessment for older people in China

**DOI:** 10.3389/fresc.2024.1384698

**Published:** 2024-04-22

**Authors:** Yuanping Zhao, Hong Xie, Kun Wang, Shouguo Liu, Juan Yan, Jianan Li

**Affiliations:** ^1^ Department of Educational Administration, Rizhao Health School, Rizhao, China; ^2^ School of Nursing, Peking University, Beijing, China; ^3^ Department of Surgery, People’s Hospital of Rizhao, Rizhao, China; ^4^ Department of Rehabilitation Medicine, The First Affiliated Hospital of Nanjing Medical University, Nanjing, China; ^5^ China Association of Social Security, Beijing, China

**Keywords:** international classification of functioning, disability and health, long-term care, disability assessment tool, disability level, classification

## Abstract

**Background:**

In the previous research, the Disability Assessment Scale based on ICF had been constructed for LTC insurance in China. To apply this scale in further studies, it is essential to establish assessment standards for disability levels.

**Objective:**

To establish standardized disability classification criteria and identify the disability statuses and levels in older people.

**Methods:**

This is a cross-sectional study, in which 1,610 older individuals in 15 long-term care institutions in China were assessed by the disability assessment scale based on ICF. Cluster analysis was used for classification of the disability levels. Mean (SD) and median (IQR) were used to describe the scores for each item and each dimension.

**Results:**

The total scores of the disability assessment scale were classified into six disability levels. The overall disability level of the 1,610 participants was moderate-to-severe. The disability in the dimension of “self-care ability and activity” was the most obvious and severe.

**Conclusion:**

The Disability Assessment Scale is capable of identifying disability statuses and levels of older people, and it can serve as a valuable tool for investigating the disabilities among old people and for conducting cross-national comparisons of disability levels.

## Introduction

The phenomenon of global population aging has been rapidly accelerating in recent years, which brings huge social and medical problems. The global population aged 65 or above was estimated at 761 million in 2021, and is projected to double by 2050, reaching approximately 1.6 billion (1). By the end of 2023, China, as the most populous country in the world, had a quarter of the global older population, which reached 217 million and accounted for 15.4% of the total population (2). As the health gradually declines with age, the number of older people with disability is gradually increasing; the number of disabled older people has now reached 40.63 million in China (3). The out-of-pocket expenses for long-term care services among disabled older people are substantial, posing unprecedented challenges to China. In response to this issue, the Chinese government implemented long-term care (LTC) insurance in 15 cities in 2016 (4). An appropriate assessment tool is the foundation for determining the correspondence between physical conditions and care services required, and could pave the way for improved LTC services (5).

Worldwide, the Barthel Index is the most widely used tool for assessing disabilities in older people (6), while there are still various assessment tools being utilized in different countries, such as the International Resident Assessment Instrument (Inter RAI) in the US (7), “Questionnaire for Identification of Nursing Care” in Japan (8), the Aged Care Funding Instrument (ACFI) in Australia (9) and the National Assessment Standard (known as the NBA) in Germany (10). However, there are several limitations in these disability assessment tools. Firstly, the Barthel Index simply applies the activities of daily living to the disability assessment of older people, lacking of comprehensive evaluation on multiple dimensions. Secondly, some of the tools contain disease diagnosis and medical treatments that not directly related to functioning or disability. Thirdly, some assessment tools, such as Inter RAI, contain excessively complex items, leading to cumbersome evaluations. Furthermore, the differences of LTC insurance policies across different countries renders these assessment tools insufficiently rigorous and applicable for direct use in disability assessment in China.

The International Classification of Functioning, Disability, and Health (ICF) is a classification of health and functioning framework (11, 12). In the ICF, functioning and disability are viewed as a complex interaction between an individual's health condition and the contextual factors of the environment, as well as personal factors (13). In the preliminary research, our team had constructed a disability assessment scale for long-term care insurance based on ICF, which showed a good level of reliability and validity (14). A scientific disability level classification standard is required for applying the disability assessment tool in China. The study aims to scientifically classify the disability levels of disabled older people with this disability assessment scale and describe the disability characteristics of the elders. After collecting disability data on older people in China, LTC service levels and contents can be reasonably distributed, and the relevant information can facilitate the development of LTC insurance standards, helping alleviate the burden of the aging population effectively.

## Methods

### Design

This cross-sectional observational study investigated older people living in 15 insurance-designated LTC institutions (including both nursing and community homecare sections) in China from April 2018 to May 2018. Using stratified sampling method, 8 pilot cities were selected considering the distribution of pilot cities in eastern, western, southern and northern China in which 2 pilot cities were randomly selected respectively. One to two representative institutions (with necessary facilities and adequate occupancy rate) were selected from each city. This study used stratified sampling to select older people from self-care and disabled areas in each institution.

### Participants

Older people were included in the study based on the following well-defined inclusion criteria: (1) aged ≥60 years; (2) resided in nursing institutions or community homecare; (3) provided informed consent. The exclusion criteria comprised inability to participate due to diseases or personal reasons. A total of 1,699 older individuals were given questionnaires and 1,610 questionnaires were returned and valid. The response rate was 94.76% and the effectiveness rate was 100%.

### Survey instrument

The Disability Assessment Scale consists of two parts: (1) General information section, including basic information such as gender and age; (2) Disability Assessment Scale section, which consists of 20 items in ICF. The score for each item is based on the Numerical Rating Scale (NRS) to determine the disability level, which ranges from 0 to 10. The higher the item scores, the more severe the disability is. Based on this, we divided the continuous scores (0–10 points) for each item into 5 levels: 0 (no problem), 1–3 (mild problem), 4–6 (moderate problem), 7–9 (severe problem), and 10 (complete problem). Specific evaluation rules for each level of each item were designed detailedly. The final score was calculated by adding up each item on the scale, which was higher in the higher level of disability in individual cases.

In the preliminary research, the Disability Assessment Scale showed good reliability and validity (14). The 20 items in the Disability Assessment Scale were divided into three dimensions: self-care ability and activity (11 items), emotion and spirit (4 items), and cognition and perception (5 items). The Cronbach's coefficient and split-half reliability of the scale were 0.969 and 0.877. The calibration validity of the scale and SF-12 were good (rPCS = −0.596, rMCS = −0.332, *p* < 0.001).

### Data collection

The older people were evaluated in the institutions by specially trained assessors. And then assessors filled in the Disability Assessment Scale on designed app. Researchers were responsible for data review and extraction.

### Data analysis

SPSS 20.0 was used for data analysis. Mean [standard deviation (SD)] and median [interquartile range (IQR)] were used to describe the scores for each item and each dimension. Cluster analysis was used for classification of the disability levels.

## Results

### Descriptive statistics

A total of 1,610 older individuals were assessed. Their ages ranged from 60 to 105 years, with a mean age of 81.5 (58.71) years. Other characteristics were shown as in Table 1.

**Table 1:** Sample characteristics (*n* = 1,610).

Project	*N* (%)
Gender	
Males	610 (37.9%)
Females	1,000 (62.1%)
Caregivers	
Children	372 (23.1%)
Spouse	61 (3.8%)
Employees	552 (34.3%)
Others	625 (38.8%)

The mean (SD) of the Disability Assessment Scale was 108.49 (54.32), the median (IQR) of the Disability Assessment Scale was 115 (60,157), and the average of the mean (SD) per item was 5.42 (2.72). It can be summarized that the overall disability level of the 1,610 older individuals was moderate–to-severe. From the perspective of the mean per item of dimensions and the sequence, the three dimensions were sorted in order as follows (from more severe to less severe): self-care ability and activity (mean per item was 5.97), emotion and spirit (mean per item was 5.00), and cognition and perception (mean per item was 4.64) (Table 2). From the perspective of the mean score of item and the sequence, the serious degrees of disability existed in “b455 Activity endurance” (7.03), “d510 Washing oneself” (6.78), “d520 Caring for one's body parts” (6.78), “d230 Daily routine” (6.57), “d450 Walking” (6.40) and “d455 Non-walking movement” (6.13), while the minor degrees of disability were “b525 Defecation” (4.82), “b280 Pain” (4.53), “d550 Eating” (4.19), “b210 Vision” (4.11) and “b230 Hearing” (4.00) (Table 3).

**Table 2:** Dimension-related results (*n* = 1,610).

Dimension	Mean (SD)	Median (IQR)	Mean (SD) per item	Sequence (mean per item from high to low)
Self-care ability and activity	65.71 (38.31)	75 (29.75,103)	5.97 (3.48)	1
Emotion and spirit	19.99 (9.05)	20 (14,26)	5.00 (2.26)	2
Cognition and perception	23.22 (14.67)	21 (11,37)	4.64 (2.93)	3

**Table 3:** Item-related results (*n* = 1,610).

Dimension	ICF code	ICF item	Mean (SD)	Median (IQR)	Sequence (mean from high to low)
Self-care ability and activity	b455	Activity endurance	7.03 (3.23)	8 (5,10)	1
	d450	Walking	6.40 (3.70)	8 (3,10)	4
	d455	Non-walking movement	6.13 (3.97)	8 (2,10)	5
	b525	Defecation	4.82 (3.93)	5 (0,9)	15
	b620	Urination	4.91 (4.01)	5 (0,9)	13
	d230	Daily routine	6.57 (3.77)	8 (3,10)	3
	d510	Washing oneself	6.78 (3.70)	8 (4,10)	2
	d520	Caring for one's body parts	6.78 (3.70)	8 (3,10)	2
	d530	Toileting	6.06 (4.07)	8 (2,10)	6
	d540	Wearing	6.04 (3.92)	8 (2,10)	7
	d550	Eating	4.19 (4.05)	3 (0,9)	17
Emotion and spirit	b130	Spirit	5.28 (2.55)	5 (3,7)	8
	b134	Sleep	5.04 (2.68)	5 (3,7)	12
	b152	Feeling	5.24 (2.71)	5 (3,7)	9
	b280	Pain	4.53 (2.93)	5 (2,7)	16
Cognition and perception	b114	Orientation	5.10 (3.87)	5 (2,10)	11
	b144	Memory	5.16 (3.67)	5 (2,9)	10
	d710	Basic interpersonal communication	4.85 (3.73)	4 (2,9)	14
	b210	Vision	4.11 (2.68)	3 (2,6)	18
	b230	Hearing	4.00(2.98)	3(2,6)	19

### Disability level results

Clustering analysis (15) was carried out on the total score of the Disability Assessment Scale. A cluster tree diagram was cut out using the 2.5 distance between classes. In our study, total disability scores were divided into five levels: level I (scores of 0–42), level II (scores of 43–92), level III (scores of 93–124), level IV (scores of 125–148), and level V (scores of 149–200), which were designated as mild, moderate, moderate-to–severe, severe, and complete disability, with a score of 0 indicting total self-care.

### Dimensions and items scores by disability level

The number and proportion of older people in different disability levels are shown in Table 4, with the largest number in level Ⅴ and the smallest number in level 0. Dimension and item scores for each disability level are shown in Tables 5, 6. As shown in Tables 5, 6, we can clearly see the differences in each dimension and item score by disability level. From level Ⅰ to level Ⅴ, the per item score of each dimension and score of each item gradually increased, indicating that as the disability level increases, the disability degree of each dimension and item in disabled elders gradually became more severe. Comparing different dimensions, in the mild and moderate disabilities, the most severe disability was reflected in the emotion and spirit aspects. While in the moderate-to-severe and more severe disabilities, the degree of disability in terms of self-care ability and activity was the most severe. Similarly, we can identify differences in the characteristics of disability items at a certain level of disability, as well as differences in disability items among individuals at different levels of disability.

**Table 4:** Number and proportion of older people in different disability levels (*n* = 1,610).

Level	0	Ⅰ	Ⅱ	Ⅲ	Ⅳ	Ⅴ
Score range	0	1–42	43–92	93–124	125–148	149–200
*n* (%)	2 (0.1)	260 (16.1)	380 (23.6)	237 (14.7)	216 (13.4)	515 (32.0)

**Table 5:** Dimension scores for different disability levels [mean (SD); median (IQR), *n* = 1,610].

Dimension		Level Ⅰ	Level Ⅱ	Level Ⅲ	Level Ⅳ	Level Ⅴ
Self-care ability and activity	Total	7.03 (7.07); 4 (2,11)	35.95 (15.38); 35 (24,47)	71.14 (13.15); 72 (61,79)	89.76 (11.41); 72 (61,79)	104.96 (6.81); 108 (102,110)
	Per item	0.64 (0.64)	3.27 (1.40)	6.47 (1.20)	8.16 (1.04)	9.54 (0.62)
Emotion and spirit	Total	12.32 (7.55); 12 (6,18)	17.80 (6.73); 18 (14,22)	18.71 (6.58); 20 (15,23)	21.52 (7.49); 20 (15,23)	26.11 (8.94); 28 (20,34)
	Per item	3.08 (1.89)	4.45 (1.68)	4.68 (1.65)	5.38 (1.87)	6.53 (2.24)
Cognition and perception	Total	6.33 (5.32); 5.5 (2,10)	13.32 (7.62); 13 (8,18)	19.30 (8.61); 19 (13,25)	26.52 (9.07); 19 (13,25)	39.55 (7.13); 41 (36,45)
	Per item	1.27 (1.06)	2.66 (1.52)	3.86(1.72)	5.30(1.81)	7.91(1.43)

**Table 6:** Items scores for different disability levels (mean (SD); median (IQR), *n* = 1,610).

Dimension	ICF item	Level Ⅰ	Level Ⅱ	Level Ⅲ	Level Ⅳ	Level Ⅴ
Self-care ability and activity	b455 activity endurance	2.12 (1.76); 2 (0.25,3)	5.39 (2.48); 5 (3,7)	7.61 (2.07); 8 (6.5,9)	8.83 (1.40); 8 (6.5,9)	9.73 (0.77); 10 (10,10)
	d450 walking	0.90 (1.34); 0 (0,2)	3.99 (2.33); 4 (2,6)	7.34 (2.40); 8 (6,10)	8.48 (1.96); 8 (6,10)	9.67 (0.99); 10 (10,10)
	d455 non-walking movement	0.55 (1.17); 0 (0,0)	3.22 (2.48); 3 (1,5)	6.90 (2.81); 7 (5,10)	8.59 (1.97); 7 (5,10)	9.73 (0.86); 10 (10,10)
	b525 defecation	0.34 (0.87); 0 (0,0)	1.76 (1.94); 2 (0,3)	4.13 (2.85); 4 (2,6)	6.62 (2.76); 4 (2,6)	8.92 (1.65); 10 (8,10)
	b620 urination	0.39 (0.98); 0 (0,0)	1.64 (1.90); 1 (0,3)	4.23 (3.01); 4 (2,6)	6.90 (2.60); 4 (2,6)	9.11 (1.48); 10 (9,10)
	d230 daily routine	0.65 (1.17); 0 (0,1)	4.01 (2.51); 3 (2,6)	7.89 (1.87); 8 (7,9)	9.00 (1.24); 8 (7,9)	9.84 (0.50); 10 (10,10)
	d510 Washing oneself	0.70 (1.27); 0 (0,1)	4.57 (2.59); 5 (3,6)	8.10 (1.61); 8 (7,10)	9.30 (0.89); 8 (7,10)	9.85 (0.53); 10 (10,10)
	d520 caring for one's body parts	0.73 (1.26); 0 (0,1)	4.55 (2.54); 5 (3,6)	8.08 (1.72); 8 (7,9.5)	9.24 (0.94); 8 (7,9.5)	9.88 (0.42); 10 (10,10)
	d530 toileting	0.23 (0.71); 0 (0,0)	2.75 (2.42); 2 (0,5)	7.12 (2.38); 8 (6,9)	8.79 (1.50); 8 (6,9)	9.85 (0.50); 10 (10,10)
	d540 wearing	0.30 (0.86); 0 (0,0)	3.13 (2.50); 3 (1,5)	6.94 (2.17); 7 (5,8)	8.56 (1.64); 7 (5,8)	9.63 (0.89); 10 (10,10)
	d550 eating	0.12 (0.48); 0 (0,0)	0.95 (1.42); 0 (0,2)	2.82 (2.51); 3 (0,5)	5.46 (3.06); 3 (0,5)	8.76 (2.05); 10 (8,10)
Emotion and spirit	b130 spirit	3.05 (2.25); 3 (1,5)	4.50 (1.97); 5 (3,5)	4.92 (1.84); 5 (4,6)	5.71 (1.99); 5 (4,6)	7.12 (2.28); 8 (6,9)
	b134 sleep	3.40 (2.30); 3 (1,5)	4.63 (2.21); 5 (3,6)	4.83 (2.20); 5 (3,6)	5.44 (2.37); 5 (3,6)	6.18 (2.98); 7 (5,8)
	b152 feeling	3.00 (2.24); 3 (1,5)	4.50 (2.04); 5 (3,6)	4.73 (2.01); 5 (4,6)	5.57 (2.38); 5 (4,6)	7.20 (2.51); 8 (6,9)
	b280 pain	2.86 (2.24); 2 (1,5)	4.18 (2.36); 4 (3,6)	4.24 (2.36); 5 (3,6)	4.77 (2.79); 5 (3,6)	5.76 (3.38); 7 (3,9)
Cognition and perception	b114 orientation	0.81 (1.33); 0 (0,2)	2.49 (2.49); 2 (0,3)	4.32 (3.05); 3 (2,7)	6.29 (2.92); 3 (2,7)	9.05 (1.64); 10 (9,10)
	b144 memory	1.13 (1.42); 0 (0,2)	2.77 (2.29); 3 (1,3)	4.38 (2.65); 4 (3,6)	6.16 (2.88); 4 (3,6)	8.93 (1.74); 10 (8,10)
	d710 basic interpersonal communication	0.72 (1.23); 0 (0,1)	2.40 (2.26); 2 (0,3)	3.91 (2.53); 3 (2,6)	5.66 (2.70); 3 (2,6)	8.86 (1.75); 10 (8,10)
	b210 Vision	2.03 (1.77); 2 (0,3)	2.94(2.03); 3(2,4)	3.36(2.09); 3(2,5)	4.48(2.40); 3(2,5)	6.23(2.32); 6(5,8)
	b230 hearing	1.64(1.90); 1(0,3)	2.72(2.24); 3(1,4)	3.33(2.30); 3(2,5)	3.94(2.61); 3(2,5)	6.47(2.58); 7(5,9)

## Discussion

The issue of population aging is a universal challenge confronting all nations and regions. However, due to the different characteristics of national regimes and policies, there is a lack of an effective tool to measure the disability level of older people among different countries and regions. This study developed the Disability Assessment Scale based on the theoretical ICF classification system, encompassing two advantages. On one hand, the ICF is a health and function classification system that uses an international standardized language code, and it can be recognized and applied throughout the world (16). In this way, our tool is a cross-nation, cross-regional and cross-cultural assessment tool which may be used to measure and compare disability levels among different countries. On one other hand, as ICF can be used across all diseases and health problems, our tool can reflect different clinical outcomes by differences of disability levels. And the results can be used as the basis for an integrated model of medicine, rehabilitation and nursing.

In this study, 20 items were selected from the ICF, covering the disabilities of the human body in the eight functional areas of active ability: self-care ability, sleep and mental status, emotion, pain, interpersonal communication, social, cognitive and sensory ability. These areas are comprehensive and essential, and may be considered an ICF core set for disabled older people. In addition, we categorized the 20 ICF items into three dimensions, each of which can be used as an assessment combination for evaluating a specific aspect of disability. ICF researchers (17) recommend using an NRS to assess the problem severity for each ICF item. The meanings represented by both extremes of the NRS scores, namely 0 and 10, are universally applicable (and similar to the meanings of 0 and 10, respectively, in the Numeric Pain Rating Scale), so an NRS can be used for all ICF items and easily understood by assessors.

Based on Disability Assessment Scale scores, we can understand the physical and mental conditions of older people. According to the overall assessment data, the disability of older people was mainly reflected in issues with self-care ability and activity. The decline of motor function is more pronounced in older individuals with disabilities, and more attention should be paid to the maintenance and training of integral motor system in disability intervention. This highlights the key points of LTC. LTC services should focus more on the comprehensive care of older people, while giving consideration to their cognitive perception regarding rehabilitation and emotional and psychological comfort.

Clustering has the advantages of not determining the number of clusters beforehand, flexibly controlling the clustering granularity, and clearly expressing the hierarchical relationship between classes. By the clustering method, the older people were divided into six disability levels based on Disability Assessment Scale scores. From level Ⅰ to Ⅴ, the mean score of each dimension and item gradually increased, which showed the feasibility of the disability level classification. Also, we can identify the disability characteristics of individuals with different levels of disability and provide targeted interventions to prevent and delay the progression of disability. In level Ⅰ and level Ⅱ, we should pay attention to the care of emotion and spirit. While in the level Ⅲ and above, we should pay more attention to the care of self-care ability and activity.

By comparing tools for the assessment and classification of LTC for older people in other countries, we can conclude that each country has a slightly different classification method based on their tool's characteristics and the country's social security levels. There are three classification methods. The first method, which is used in Japan and the US, involves a rating system software where the assessment results are entered and it can display the disability level simultaneously. For example, Japan's Department for LTC Insurance (8) designed a rating system software to identify the level of care requirements based on “Questionnaire for the Identification of Nursing Care”. The care requirements are divided in to three levels: self-reliant, support required (type 1 or 2) and care required (type 1, 2, 3, 4, 5). The second method, which is used in Germany, is to add up the total scores of each dimension, taking into account the weight of each dimension. Germany (10) weights each dimension of NBA as follows: dimension 1 accounts for 10%, dimension 2 and 3 account for 15%, dimension 4 accounts for 40%, dimension 5 accounts for 20%, and dimension 6 accounts for 15%. According to the total score, the older people are divided into five levels of disability: 15–29, 30–49, 50–59, 60–89 and ≥90, which are designated independent level 1–5 respectively. The third method, which is used in Australia, is to combine the results of each dimension to obtain the disability level of older people without weighting. Australia (9) designed each dimension of ACFI so it can be divided into H, M, and L according to the total score for the items of each dimension. The combination of the three levels in three dimensions divide disabled old people into a high or low care level. The classification method of the Disability Assessment Scale in this study belongs to the second category and does not consider the weight of each dimension, which makes it more direct and time-efficient when determining disability levels.

The ICF-based Disability Assessment Scale can be used as a screening tool for older people in China. It is well known that China has a large number of older people and a poor level of social security. The tool was designed with a simple structure to facilitate basic screening of functional levels in older people in China, aiming to optimize the allocation of LTC resources in a more rational and efficient manner. Based on the Disability Assessment Scale, the next step is to design a more detailed assessment tool. In previous surveys of the disability in China, such as the Chinese Urban and Rural Older People Tracking Survey, Chinese Follow-Up Investigation on Influential Factors of Longevity and Health, Chinese Health and Old-Age Care Survey, and the Seventh National Population Census, the assessments were solely focused on the performance of activities of daily living (18). In contrast, the Disability Assessment Scale in this study is more comprehensive and standardized, making it more practical for conducting large-scale investigations.

Also, the ICF-based tool may be used to assess disability across national populations. It is well known that the International Classification of Diseases (ICD) (19) is the international unified disease classification method published by the WHO. The ICD standardizes disease names and helps to make disease information as widely shared as possible in order to reflect countries’ health situations. After issuing the ICD, the WHO issued the ICF as the international unified functioning classification system. The ICF and ICD are the two major health classification systems published by the WHO. The ICD can only reflect the classification of etiological diagnoses such as disease and injury, but these diseases may increase the risk of disability after clinical treatment, causing additional expenses that cannot be compensated by medical insurance. The ICF can be used to reflect post-acute and convalescent disability and it promotes the linkage of medical care and LTC, the medical maintenance and the realization of an integrated health strategy. Due to health conditions, older people may be sent to different care institutions, such as hospitals, nursing homes and convalescent homes where elders may face repeat assessments. Using the Disability Assessment Scale, the information can be accessed with the agreement of individuals, and a people-centered care system will be promoted.

In the field of disability assessment, the international community used to focus more on the decline of physical function when evaluating disability in elders, which was particular evident in the assessment of the Barthel index. In recent years, researchers have gradually focused on various aspects of disability such as cognitive function, contributing to the development of comprehensive assessment tools. The decline in physical function among older individuals is more obvious and receive more social attention, while the cognitive decline such as Alzheimer's disease is initially coming into people's mind. The weight of physical and cognitive functions should be continuously adjusted according to national conditions, changes in the spectrum of diseases, and other complex factors.

The Disability Assessment Scale can describe the overall level of disability among older people, and long-term care insurance can provide protection for some disabilities based on their disability levels. However, specific populations, such as elders with only cognitive disability but good physical function, may not be covered by long-term care insurance. Therefore, special attention needs to be paid to this group. One possible solution is to build an additional pathway for dementia assessment to evaluate cognitive function, which can come to an additional score as a supplementary assessment. If the elders failed to be evaluated as severe or complete disability in the first-round assessment, they can be also considered as severely dementia elders with severe problems both in “b114 Orientation” and “b144 Memory” (score ≥7 points). The limitation of the research is that the existing data cannot verify the rationality of grouping. Therefore, we are collecting data on care services of disabilities in current research and the rationality of grouping disabilities will be verified through the different burden of care needed.

## Study limitations

In this study, the sampling method and sample size represent study limitations. In a future study, we need to conduct stratified and phased sampling, using stratification variables such as the number of beds in each LTC institution and the urban characteristics of the cities where the LTC institutions are located.

## Conclusion

In this study, the Disability Assessment Scale developed based on the ICF is capable of identifying the disability status and classifying the level of disability in older individuals. Based on the assessment scores of the tool, the LTC insurance funds and other resources in China can be reasonably distributed. At the same time, the tool is internationally standardized and simple, and can therefore be used for large-scale disability investigations in China and comparisons of older persons’ disability levels between countries.

## Data Availability

The original contributions presented in the study are included in the article/Supplementary Material, further inquiries can be directed to the corresponding authors.
